# Unveiling Mixed Cryoglobulinemia in Suspected Sepsis Without a Source

**DOI:** 10.7759/cureus.57684

**Published:** 2024-04-05

**Authors:** Eder Luna-Ceron, Lakshmi Kattamuri, Katherine Vidal, Guillermo de Jesus Aguirre-Vera, Angelica Lehker

**Affiliations:** 1 Internal Medicine, Texas Tech University Health Sciences Center El Paso, El Paso, USA; 2 Clinical Sciences, Escuela de Medicina y Ciencias de la Salud Tecnologico de Monterrey, Mexico, MEX

**Keywords:** rheumatoid factors, direct-acting antivirals, bacterial sepsis, hepatitis c, mixed cryoglobulinemia

## Abstract

Cryoglobulinemia is an uncommon condition characterized by the presence of cryoprecipitable immune complexes in circulation, leading to clinical symptoms like purpura, muscle weakness, and joint pain. Specifically, mixed cryoglobulinemia involves the formation of these complexes due to rheumatoid factors, mainly IgM, occasionally IgG or IgA. Previously, Hepatitis C (HCV) was a common cause of mixed cryoglobulinemia, as the chronic HCV infection triggered immune responses that resulted in cryoglobulin formation. However, the emergence of direct-acting antivirals (DAAs) for HCV treatment has shifted the landscape, with autoimmune and lymphoproliferative disorders becoming more prominent etiological factors for mixed cryoglobulinemia. This case report features a 67-year-old woman with a history of Hepatitis C-related cirrhosis. She presented at the emergency department with signs of septic shock and widespread joint pain, particularly in the knees, shoulders, and neck. Effective sepsis management was achieved using antibiotics, albumin infusion, and midodrine. Nonetheless, significant cervical and bilateral knee pain persisted. Further examination uncovered hypocomplementemia and positive results for rheumatoid factors (IgA, IgM, IgG) and cryoglobulin agglutination, confirming the diagnosis of mixed cryoglobulinemia. This case emphasizes the importance of considering mixed cryoglobulinemia in chronic Hepatitis C patients displaying fatigue and joint pain, even in the absence of the traditional clinical manifestations. Moreover, the case underscores the dual benefits of DAA treatment for Hepatitis C in individuals with mixed cryoglobulinemia by achieving viral eradication and alleviating cryoglobulinemia-related symptoms, thus preventing further organ damage.

## Introduction

Cryoglobulinemia refers to a condition characterized by the presence of circulant serum cryoglobulins, a subset of immunoglobulins that can precipitate when exposed to temperatures lower than 37°C [1]. Brouet et al. classified cryoglobulinemia into three types based on immunoglobulin composition [2]. In this regard, type I cryoglobulinemia, or simple cryoglobulinemia, is the result of monoclonal immunoglobulin, usually immunoglobulin M (IgM) and rarely immunoglobulin (IgA) or immunoglobulin G (IgG) [3]. On the other hand, type II and type III cryoglobulinemia, also known as mixed cryoglobulinemia (MC) is characterized by the presence of both monoclonal and polyclonal immunoglobulins, typically IgM and, occasionally, IgG or IgA [1,3]. Regarding type II MC, cryoglobulins are comprised of both monoclonal and polyclonal IgG or IgM. Type III MC immunoglobulins are comprised only of polyclonal IgG and IgM [1,3]. Importantly, in MC, cryoglobulins can work as rheumatoid factors (RFs), which means that these immunoglobulins can attach to other antibodies, resulting in circulant immunocomplexes [1]. Additionally, cryoglobulinemia can be classified regarding underlying disorders, for example, cryoglobulinemia not linked to any specific disease has been referred to as essential cryoglobulinemia [1]. On the other hand, cryoglobulinemia associated with a particular disease is known as secondary cryoglobulinemia [4].

Cryoglobulinemia is considered a rare disease, affecting less than five cases per 10,000 individuals, affecting more females than males according to a large epidemiological study [5]. Patients developing cryoglobulinemia classically manifest symptoms associated with immunocomplex deposits in peripheral vasculature including purpura, livedo reticularis, peripheral neuropathy, weakness, and arthralgias [1,4]. The presence of type I cryoglobulinemia is usually associated with an underlying lymphoproliferative disease such as non-Hodgkin B cell lymphoma (NHBL) [6]. On the other hand, MC types II and III have a significant association with underlying hepatitis C virus (HCV) infection and other autoimmune disorders such as systemic lupus erythematosus (SLE), Sjögren’s syndrome, and rheumatoid arthritis [1,4].

It has been extensively reported that aberrant production of autoantibodies and abnormalities that result in increased proliferation and transformation of B cells is the cornerstone pathophysiology for the development of MC [1]. In this regard, it has been shown that the immune response triggered by chronic HCV infection leads to the formation of cryoglobulins, causing vasculitis and various clinical manifestations such as skin lesions, joint pain, and kidney involvement [3]. Before the advent of direct-acting antivirals (DAAs), HCV infection was a common cause of MC [7]. However, with the introduction of DAAs for the treatment of HCV, the landscape of mixed cryoglobulinemia has seen an etiological shift toward autoimmune and lymphoproliferative disorders, HCV-related MC treated with DAA has shown dramatic amelioration and prevented further organ damage [7]. In this article, we summarize the clinical course and diagnostic approach of a case of MC type III secondary to HCV infection.

## Case presentation

We present the case of a 67-year-old Hispanic female with a medical history of cirrhosis secondary to HCV, hypertension, and a recent percutaneous cholecystostomy placement secondary to acute cholecystitis three weeks before this presentation. Importantly, she reported a prior complicated cesarean section in the 1980s that required multiple blood transfusions resulting in acquiring HCV. Importantly, the patient had a confirmed diagnosis of HCV 30 years after the source exposition. Additionally, the patient was referred before management with glecaprevir-pibrentasvir but abruptly discontinued the treatment on her own after four weeks because of jaundice and rash. The patient presented with a two-week history of generalized weakness, profound fatigue, fever, vomiting, and worsening of preexisting chronic bilaterally symmetrical joint pain in knees, shoulders, and cervical spine. She referred to self-medication with over-the-counter analgesics and oral corticosteroids with partial improvement of joint pain. She discontinued the use of these medications three weeks before this admission. Upon admission, the patient denied any cough, dysuria, hematuria, diarrhea, or any contact with a sick relative.

Upon admission, the patient was found febrile (38.9°C), tachycardic (115 beats per minute), and hypotensive (61/38 mmHg) with significantly altered mental status. Physical examination was remarkable for icteric conjunctiva, poor dentition, painful range of motion of neck, knees bilaterally, and cervical spine. The patient was found also with a significantly distended abdomen, which was tender, and a right abdominal upper quadrant biliary drain with no signs of bleeding or surrounding erythema. Physical examination was also remarkable for several petechiae on the extensor surfaces of both the upper and lower limbs.

The laboratories and paraclinical investigations observed upon admission are shown in Table 1.

**Table 1:** Laboratories and paraclinical investigations along with the hospitalization of the patient. Data are presented as the units shown in the parameter column. Abbreviations: WBC: white blood cell count; Neu: neutrophil count, Hb: hemoglobin, PLT: platelet count; INR: international normalized ratio; Na: sodium, K: potassium; Cr: creatinine; AST: aspartate aminotransferase; Alk Phos: alkaline phosphatase; ALT: alanine aminotransferase; LA: lactic acid, CRP: C-reactive protein, IgG: immunoglobulin G

Parameter	Admission (7/15/23)	7/17/2023	7/19/2023	7/20/2023	Reference range
WBC (Ux10^9^/L)	27.63	14.3	7.16	6.73	4.5 to 11.0 x 10^9^/L
Neu (Ux10^9^/L)	19.62	10.48	3.74	3.41	2.5 to 6 x 10^9^/L
Hb (g/dL)	10.3	9.5	10.6	10.3	12.0 to 16.0 g/dL
PLT (Ux10^9^/L)	210	178	186	171	150 to 400 x 10^9^/L
INR (IU)	1.2				0.8 to 1.2 IU
Na (mmol/L)	133	137	136	135	135 to 145 mmol/L
K (mmol/L)	5.8	4.0	3.6	3.9	3.5 to 5.2 mmol/L
CO_2_ (mmol/L)	13	18	23	22	23 to 29 mmol/L
Cr (mg/dL)	1.7	0.7	0.5	0.4	0.7 to 1.3 mg/dL
Bilirubin (mg/dL)	2.8	2.6	1.4	1.6	0.1 to 1.2 mg/dL
Albumin (g/dL)	3.3	2.1	2.3	2.4	3.4 to 5.4 g/dL
AST (U/L)	36	26	42	40	10 to 36 units/L
Alk Phos (U/L)	97	73	87	79	44 to147 U/L
ALT (U/L)	16	10	17	17	4 to 36 U/L
LA (mmol/L)	2.4	1.3	0.8	0.2	<2 mmol/L
CRP (mg/dL)	5.1	2.3	1.02	0.8	0.3 to 1.0 mg/dL
Hepatitis C IgG	Positive				Negative

Importantly, the patient was found to have neutrophilic leukocytosis, mild hyponatremia, direct hyperbilirubinemia, transaminitis, hyperlactatemia, acute kidney injury (AKI), and elevated acute phase reactants. In this regard, the patient was initially managed along the lines of severe sepsis with intravenous fluid resuscitation and broad-spectrum antibiotics (vancomycin and piperacillin-tazobactam). Blood and urine cultures taken at admission and repeated subsequently did not show growth of any microorganisms. A respiratory BioFire panel (BioFire Diagnostics, Salt Lake City, Utah) was negative for any viral and bacterial microorganisms. Diagnostic paracentesis was performed showing changes compatible with portal hypertension, but there was no evidence of spontaneous bacterial peritonitis.

**Figure 1:**
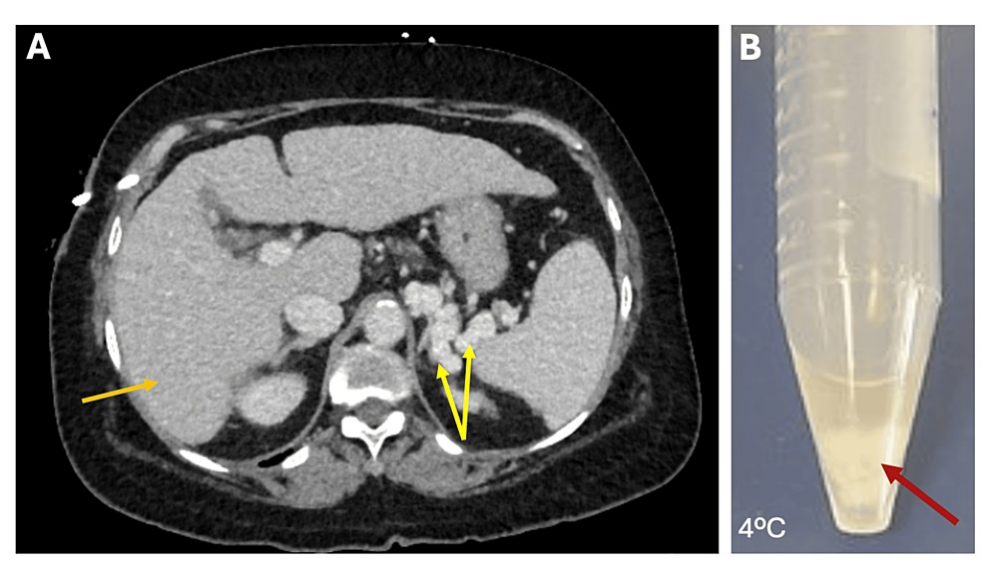
Representative images from abdominal CT and serum sample showing a characteristic cryocrit formation. A. Abdominal CT showed the presence of the liver with cirrhotic morphology (orange arrow), as well as with multiple left upper quadrant varices consistent with portal hypertension (yellow arrows). B. Serum sample showing a characteristic cryoglobulin pellet present at 4°C (red arrows).

CT of the maxillofacial, thorax, and abdomen were unremarkable for any apparent infectious source. However, abdominal CT showed the presence of a liver with cirrhotic morphology, as well as multiple left upper quadrant varices consistent with portal hypertension (Figure 1A). The Hepatitis panel was positive for HCV IgG. After 24 hours of antibiotic therapy and fluid resuscitation, the patient's leukocyte counts, inflammatory markers, and AKI improved (Table 1). However, she remained hypotensive, requiring midodrine to maintain her mean arterial pressure (MAP). After 48 hours, the patient was able to be weaned from midodrine.

Importantly, the patient continued to experience ongoing joint pains and fatigue. The imaging of the cervical spine, knee, and ankles failed to reveal any abnormality. Considering her long history of joint pains, she was worked up for potential systemic autoimmune and rheumatological diseases, which revealed a positive antinuclear antibody (ANA), RFs, and low serum complement (C4) levels, as shown in Table 2. Considering her medical history of hepatitis C infection, her present clinical symptoms, and the results of laboratory tests, suspicion of mixed cryoglobulinemia has arisen. Further approach revealed the presence of a positive cryocrit (Figure 1B). Importantly, immunoblotting tests showed the presence of polyclonal IgA, IgM, and IgG rheumatoid factor subsets (Table 2). With this evidence, the diagnosis of type III MC was made.

**Table 2:** Serum immunological parameters employed for diagnosis of mixed cryoglobulinemia. Data are presented as the units shown in the parameter column. Dilutions are shown as a ratio. Abbreviations: ANA: Antinuclear antibodies; IgA: immunoglobulin A, IgG: Immunoglobulin G; IgM: immunoglobulin M. IU: International units

Parameter	Result	Reference range
Complement 3 factor (mg/L)	95	75 to 175 mg/dL
Complement 4 factor (mg/L)	13.8	20 to 45 mg/dL
ANA titer (maximal dilution)	

Positive (1:1280)	<1:160
Cryoglobulin Presence at 4°C	Positive	Negative
Rheumatoid factor IgA titer (maximal dilution)	Positive (>1:100)	<1:80
Rheumatoid factor IgG titer (maximal dilution)	Positive (>1:100)	<1:80
Rheumatoid factor IgM titer (maximal dilution)	Positive (>1:100)	<1:80

In the absence of organ-threatening manifestations after seven days of admission, the patient was discharged and referred to outpatient rheumatology and gastroenterology for further management.

## Discussion

We presented the case of a female patient with findings of sepsis without an identified source and severe articular pain with immunological studies suggesting type III MC. This case depicts a case of MC with multisystemic involvement including musculoskeletal, neurological, and skin implications. In this regard, MC is characterized by the presence of a triad, also known as Meltzer’s triad, which comprises purpura, weakness, and arthralgias [1]. This case did not show significant purpura; however, the presence of weakness and arthralgias was significant. In agreement with an Italian cohort of patients with MC, arthralgias and weakness were the two most reported clinical presentations [8]. In this regard, it has been reported that MC has other important clinical features, including peripheral neuropathy, vasculitis-associated skin ulcers, renal disease, and liver involvement [4]. Remarkably, in our patient, there was significant peripheral neuropathy, given the findings from the physical examination that showed bilateral lower-limb sensation impairment. Interestingly, because of its diverse clinical presentation, type III MC can manifest with only one or two apparent or clinically predominant features, leading to a difficult or delayed diagnosis [9]. This observed variability can be attributed to differences in patient recruitment across various specialist centers, as well as variations in the racial composition among patient cohorts [9]. In this case, given the patient's autoimmune markers, including positive ANA, RFs, and low complement levels, suspicion of an underlying autoimmune process was raised.

Type III MC is characterized by the presence of polyclonal IgG, IgM, and complement components within the cryoglobulin complexes, which can deposit in various tissues and organs, leading to systemic manifestations [1]. In the case of our patient, these findings were evident as serology showed elevated levels of IgM, IgA, and IgG RF subtypes raising concerns about polyclonal involvement. Positive cryoglobulins directed our diagnosis suspicion for type III MC. The diagnosis of MC can also be helped by the performance of a skin biopsy showing the characteristic leukocytoclastic vasculitis involving medium- and, more often, small-sized blood vessels [1]. However as described before, in the presence of serology and clinical features suggesting MC, the diagnosis can be done not requiring histological evidence [9].

As HCV infection is the primary underlying cause of MC in most cases, individuals may develop overt chronic hepatitis, typically exhibiting a mild-to-moderate clinical course, at any point during the disease's natural progression [9]. Although the development of cirrhosis has only been seen in about 25% of the patients with MC [8], other factors such as chronic alcohol consumption may be associated with the development of cirrhosis in this case. In this regard, it has been proposed that HCV may lead to chronic immune stimulation by the expression of immune-reactive proteins as the core protein, leading to the development of cross-reactive autoantibodies, autoreactive T-cells, and aberrant lymphoproliferation leading to the deposit of immunocomplexes [10]. Interestingly, these events may also be associated with the development of other autoimmune disorders that have been linked to MC such as thyroiditis, lung fibrosis, or the development of diabetes [9,10]. In the case of our patient, these events may explain her long history of hypothyroidism.

There is evidence suggesting that DAAs have significantly improved the condition of patients with HCV-related MC, preventing further organ damage [11]. Treating HCV with DAAs not only eliminates the virus but also resolves symptoms associated with cryoglobulinemia and prevents additional organ damage, thus enhancing quality of life [11]. Failure to treat HCV may have been a primary factor in the development of MC as a serious complication of chronic HCV in our patient. Therefore, future management strategies should prioritize addressing both HCV and MC simultaneously.

Managing MC syndrome is challenging because of its complex causes and varied clinical presentation. As HCV is the primary cause, efforts should focus on eradicating it because of its chronic stimulation of the immune system [9]. It is crucial to exercise caution when considering the use of alpha-interferon in MC patients, as it can exacerbate symptoms [9]. While evidence suggests that DAAs are preferable for MC patients, there is still a need for robust clinical trials to assess their efficacy [11].

Life-threatening complications of MC, such as membranoproliferative glomerulonephritis and severe neuropathy, may require interventions like plasmapheresis and immunosuppressive therapies such as rituximab alongside DAA treatment [1,4,9]. These interventions aim to reduce B-cell expansion and the production of new autoantibodies, ultimately managing and preventing cryoglobulinemic vasculitis [1,4,9].

Treatment for MC should be individualized based on the patient's specific symptoms, with severe vasculitis manifestations requiring prompt treatment with high doses of steroids, plasma exchange, or immunosuppressive agents like cyclophosphamide or rituximab [1,4,9]. As our patient did not exhibit severe vasculitis symptoms, a detailed and tailored outpatient treatment plan was deemed appropriate.

## Conclusions

In conclusion, it is imperative to maintain a high index of suspicion for MC in patients with chronic HCV infection, particularly when presenting with symptoms such as fatigue and arthralgias, even in the absence of Meltzer’s triad. The clinical complexity of MC necessitates thorough evaluation, especially considering the evolving epidemiological landscape shaped by the widespread use of DAAs, which has brought autoimmune and lymphoproliferative disorders to the forefront as emerging etiological factors for MC. Therefore, patients with suspected MC warrant comprehensive workup for timely diagnosis and the identification of associated disorders at presentation. For those with concurrent chronic HCV infection and MC, treatment with DAAs offers not only viral eradication but also alleviation of cryoglobulinemia-related symptoms and prevention of further organ damage, thereby enhancing quality of life. In cases where severe vasculitis manifestations are present, prompt intervention with high-dose steroids, plasma exchange, and/or immunosuppressive agents like cyclophosphamide or rituximab is essential. This case underscores the evolving landscape of MC and emphasizes the necessity of a multidisciplinary approach to its diagnosis and management, focusing on tailored, personalized care to meet the unique needs of each patient.
